# Mitochondrial pathogenic mutations are population-specific

**DOI:** 10.1186/1745-6150-5-68

**Published:** 2010-12-31

**Authors:** Michael S Breen, Fyodor A Kondrashov

**Affiliations:** 1 Bioinformatics and Genomics Programme, Centre for Genomic Regulation, UPF, C/Dr. Aiguader 88, Barcelona Biomedical Research Park Building, 08003 Barcelona, Spain

## Abstract

**Background:**

Surveying deleterious variation in human populations is crucial for our understanding, diagnosis and potential treatment of human genetic pathologies. A number of recent genome-wide analyses focused on the prevalence of segregating deleterious alleles in the nuclear genome. However, such studies have not been conducted for the mitochondrial genome.

**Results:**

We present a systematic survey of polymorphisms in the human mitochondrial genome, including those predicted to be deleterious and those that correspond to known pathogenic mutations. Analyzing 4458 completely sequenced mitochondrial genomes we characterize the genetic diversity of different types of single nucleotide polymorphisms (SNPs) in African (L haplotypes) and non-African (M and N haplotypes) populations. We find that the overall level of polymorphism is higher in the mitochondrial compared to the nuclear genome, although the mitochondrial genome appears to be under stronger selection as indicated by proportionally fewer nonsynonymous than synonymous substitutions. The African mitochondrial genomes show higher heterozygosity, a greater number of polymorphic sites and higher frequencies of polymorphisms for synonymous, benign and damaging polymorphism than non-African genomes. However, African genomes carry significantly fewer SNPs that have been previously characterized as pathogenic compared to non-African genomes.

**Conclusions:**

Finding SNPs classified as pathogenic to be the only category of polymorphisms that are more abundant in non-African genomes is best explained by a systematic ascertainment bias that favours the discovery of pathogenic polymorphisms segregating in non-African populations. This further suggests that, contrary to the common disease-common variant hypothesis, pathogenic mutations are largely population-specific and different SNPs may be associated with the same disease in different populations. Therefore, to obtain a comprehensive picture of the deleterious variability in the human population, as well as to improve the diagnostics of individuals carrying African mitochondrial haplotypes, it is necessary to survey different populations independently.

**Reviewers:**

This article was reviewed by Dr Mikhail Gelfand, Dr Vasily Ramensky (nominated by Dr Eugene Koonin) and Dr David Rand (nominated by Dr Laurence Hurst).

## Background

The discovery of genetic variants associated with human diseases is widely anticipated to be one of the stepping stones leading to an era of personalized medicine. Hundreds or even thousands of deleterious alleles segregate in the human population [1-6] and contribute to a vast diversity of disease conditions [7,8]. While most of them are individually only slightly deleterious [2] taken together an average genome carries several lethal equivalents [6,9]. In principle, correlating genetic variants to disease phenotypes in a sample of the human population can reveal those variants that are likely to contribute to disease. This is now routinely attempted by genome-wide association studies (GWAS) [10-15] or deleterious alleles predicted computationally on the back of large-scale sequencing efforts [2,4]. However, the success of GWAS in particular is dependent on pathogenic polymorphisms segregating at relatively high frequency in the population [12,13,16,17].

If common diseases are caused by common variants then the polymorphisms implicated by GWAS are likely to contribute to disease not only in the sample from the study but also in a relatively large fraction of individuals with the disease phenotype in the entire human population. However, if common diseases in the human population are caused by many rare variants then the probability of discovery of these variants is low [12,13] and different populations are likely to carry different variants associated with one disease. Since a majority of GWAS are performed within specific human populations [11,18,19] it is currently unclear if the disease variants identified by a study as major contributors to a specific disease in one population also contribute to the same pathology in a different population.

To study the population-specific distributions of SNPs we performed a comparison of variation encoded in the mitochondrial genome in African and non-African populations. We focused on the mitochondrial genome for three reasons. First, the mitochondrial genome contributes to dozens of genetic pathologies [8,20], second, it has not been subject to a genome-wide survey of segregating deleterious polymorphism and third, the diversity of available completely sequenced mitochondrial genomes allowed us to consider genomes from different populations independently.

## Methods

### Genomic data

We obtained complete mitochondrial genome sequences from GenBank using "complete genome AND Homo sapiens [orgn]" as a query with "Mitochondrion and Genomic DNA/RNA" selected in the Limits section of the nucleotide search [21]. From this dataset we excluded all genomes that were sequenced from an individual with a known pathological condition as reflected in the GenBank file leaving a total of 4458 genomes. We identified 401 genomes as belonging to L haplotypes (African) and 4057 genomes as belonging to the N or M haplotype (non-African). Most of these genomes were already assigned to these haplogroups. For the remaining genomes we identified their haplogroup via BLAST searches. We then made a multiple alignment of all genomes using the MEGA 4 program package [22] with manual curation. In this alignment we identify polymorphic sites, those sites in which more than one nucleotide allele is found. Of these alleles we identify the minor alleles, those that are the least frequent at a polymorphic site, in protein coding, tRNA and rRNA genes (Table 1).

**Table 1:** All polymorphism data from African and non-African populations.

	Number of variable sites			Number of minor alleles	
SNP Category	Total	African	Non-African	African	Non-African

Synonymous	2574 (67.1%)	792 (71.0%)	2422 (67.3%)	8614	39183

- Four fold	1177 (45.7%)	419 (53.0%)	1274 (52.6%)	4877	20284

Nonsynonymous	1263 (32.9%)	323 (29.0%)	1178 (32.7%)	2942	15688

- Unknown	4 (0.3%)	1 (0.3%)	4 (0.3%)	5	51

- Benign	922 (73.0%)	263 (81.4%)	865 (73.4%)	2727	14611

- Damaging	338 (26.7%)	59 (18.3%)	309 (26.3%)	210	1025

Pathogenic total	87	27	85	83	1259

- Coding regions	55	19	53	56	956

- tRNAs	25	7	25	26	177

- rRNAs	7	1	7	1	126

Some polymorphic sites were shared between the two populations, leading to fewer polymorphic sites in the total category than just the sum of polymorphic sites from each population.

### Polymorphism data

Polymorphisms were classified for each protein coding gene into "benign", "possibly damaging" and "probably damaging" using a standalone version of PolyPhen 2 [23] with the "possibly damaging" and "probably damaging" categories pooled into one "damaging" category for the purpose of our analysis. PolyPhen 2 normally utilizes distant sequences for its prediction and does not accept more than 1000 homologues in the alignment used for classifying SNPs into the three categories. For mitochondrial proteins more than 1000 homologues were typically available and PolyPhen 2 did not always select the most closely related orthologues for the alignment. We thus ran PolyPhen 2 using alignments of all primate orthologous proteins. Although we treated "probably damaging" and "possibly damaging" as a single category, when both categories were compared our results and conclusions remained the same (data not shown). We estimated nucleotide diversity (π), which is the average fraction of sites occupied by different alleles in all pairwise sequence comparisons in the sample [24], using MEGA 4 [22] with pairwise deletion and selecting the Nei-Gojobori method to estimate the number of substitutions between sequences. We obtained data on pathogenic mutations from the MitoMap web resource [25] and to reduce the possibility of erroneous pathogenic mutations affecting our results we excluded all categories of pathogenic mutations other than "Reported" and "Confirmed" as well as all mutations reported by [26]. Mann-Whitney U-test was applied to test the statistical significance of the differences reported in the tables, with the values in Table 3 obtained by the Monte Carlo sampling by 1000 replicates of 401 sequences selected from the non-African genomes for the analysis. As there is a large difference between the African and non-African sample sizes in our dataset we applied a Monte-Carlo technique to obtain sample-independent estimates when required. Values in Tables 2-4 are reported with standard errors.

**Table 2:** π for different types of sites.

SNP Category	African	Non-African	U-test p-value
Synonymous	0.010 ± 1 × 10^-5^	0.0054 ± 1 × 10^-6^	< 1 × 10^-5^

- Four fold	0.0089 ± 1 × 10^-5^	0.0044 ± 8 × 10^-7^	< 1 × 10^-5^

Nonsynonymous	0.0011 ± 2 × 10^-6^	0.00087 ± 1 × 10^-7^	< 1 × 10^-5^

**Table 3:** Average frequency of minor alleles.

SNP Category	African (401 genomes)	Non-African (4057genomes)	Non-African (Monte-Carlo sampling of 401 genomes)	U-test p-value
Synonymous	0.027 ± 0.003	0.0040 ± 0.0004	0.011 ± 4 × 10^-6^	7.9 × 10^-6^

- Four fold	0.029 ± 0.004	0.0039 ± 0.0005	0.011 ± 4 × 10^-6^	2.4 × 10^-6^

Nonsynonymous	0.023 ± 0.005	0.0033 ± 0.0006	0.011 ± 4 × 10^-6^	1.5 × 10^-6^

- Benign	0.026 ± 0.006	0.0042 ± 0.0008	0.012 ± 5 × 10^-6^	1.0 × 10^-6^

- Damaging	0.0089 ± 0.009	0.00082 ± 9 × 10^-5^	0.0038 ± 3 × 10^-6^	1.7 × 10^-3^

Pathogenic total	0.0077 ± 0.003	0.0037 ± 0.001	0.0084 ± 8 × 10^-6^	1.0 × 10^-6^

- Coding regions	0.0074 ± 0.003	0.0044 ± 0.001	0.0095 ± 1 × 10^-5^	1.0 × 10^-6^

## Results

Differences in the level of polymorphism among the African and non-African population have been studied extensively for the nuclear genome [27-31]. Thus, some of the data reported here, and their interpretation, are analogous to those reported for the nuclear genome. In agreement with previously published data [27-31] the African genomes showed higher nucleotide diversity, π, at all classes of sites compared with non-African genomes (Table 2), with this difference being less pronounced for nonsynonymous SNPs (nSNPs). The nucleotide diversity obtained for the mitochondrial genes was higher than that for equivalent sites in the nuclear genome with mitochondrial synonymous diversity (π_s_) ~2.5 fold and mitochondrial nonsynonymous diversity (π_n_) ~6.5-8.5 fold higher than the estimates from the nuclear genome (Table 1 from ref. [1]), which is consistent with a higher rate of mutation in the mitochondrial genome [32] that allowed nucleotide diversity to accumulate faster after the recent population expansion [27-31]. The larger difference between π_s _and π_n _indicates that nonsynonymous sites are under stronger selection in the mitochondrial genome [33,34]. However, while the difference in mitochondrial and nuclear π_s _was similar for African and non-African populations the difference for π_n _was lower for the non-African population (8.5 fold in the African and 6.5 for non-African genomes), indicating that negative selection against mitochondrial nonsynonymous alleles has been relaxed in the non-African population.

We used PolyPhen 2 [23] to predict the fitness impact of nSNPs classifying each nSNP as either "benign" or "damaging" (see Methods). The damaging category must be enriched for SNPs that are likely to be deleterious, while the benign category includes likely neutral variants [2,4,23], as is indicated by a 2-5 fold lower average frequency of SNPs labelled as damaging compared to the frequency of those estimated to be benign (Table 3). Consistent with our data on nucleotide diversity, we found alleles in the African population to have a higher average frequency than in the non-African population. Congruent results were obtained when measuring the average number of minor alleles per genome, with African genomes carrying approximately twice the number of minor alleles, with this difference being similar for benign and damaging SNPs (Table 4).

**Table 4:** Average number of minor alleles per genome.

SNP Categories	African (401 genomes)	Non-African (4057 genomes)	U-test p-value
Synonymous	21.5 ± 0.5	9.7 ± 0.1	2.2 × 10-6

- Four fold	12.2 ± 0.3	5.0 ± 0.06	2.2 × 10-6

Nonsynonymous	7.3 ± 0.2	3.9 ± 0.04	2.2 × 10-6

- Benign	6.8 ± 0.2	3.6 ± 0.03	2.2 × 10-6

- Damaging	0.53 ± 0.04	0.25 ± 0.009	2.2 × 10-6

Pathogenic total	0.21 ± 0.02	0.31 ± 0.008	1.3 × 10-6

- Coding Regions	0.14 ± 0.02	0.24 ± 0.007	1.6 × 10-3

Levels of polymorphism are influenced by mutation, selection and genetic drift. All three of these factors necessarily need to be invoked to explain all of the observations mentioned above. First, the higher π_n _and π_s _in the mitochondrial relative to the nuclear genome is consistent with a higher rate of mutation in the mitochondrial genome [32]. Second, the larger difference between π_n _and π_s _in the mitochondrial genome relative to the nuclear genome indicates that nonsynonymous sites in the organelle are under stronger negative selection. Finally, a largely similar difference in π_n _and π_s _and in the average number of minor alleles per genome between the African and non-African populations indicates that genetic drift has been a stronger factor in shaping the difference between the levels of polymorphism in African and non-African populations than differences in selection pressure. However, a slight difference in the strength of negative selection in the African versus non-African population is also consistent with these and nuclear data [4].

**Figure 1:**
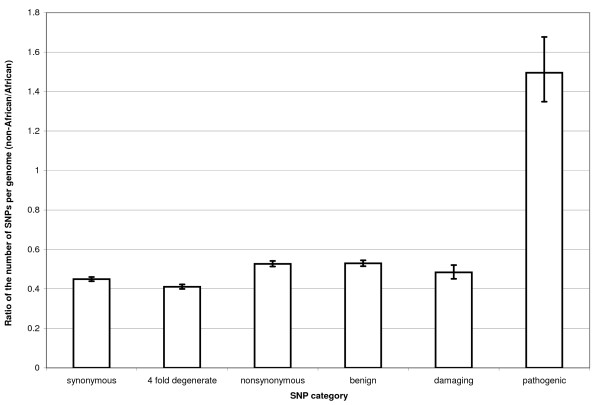
**Ratio of the average number of minor alleles per genome of the two populations**. For each category of polymorphisms we obtained the ratio by dividing the average number of minor alleles per genome in the non-African population by the average number of minor alleles per genome in the African population. Data shown with s.e.m.

**Figure 2:**
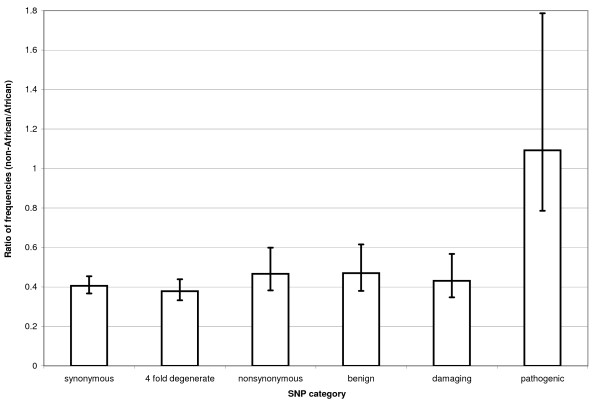
**Ratio of the average frequency of segregating minor alleles of the two populations**. For each category of polymorphisms we obtained the ratio by dividing the average frequency of segregating minor alleles per genome in the non-African population by the average frequency of minor alleles per genome in the African population. Data shown with s.e.m.

Using data from MitoMap [25] we then identified those minor alleles among our dataset that are known to contribute to genetic pathologies. The pathogenic SNPs show the opposite trends when comparing African and non-African population than all other types of polymorphism. Pathogenic SNPs have a higher frequency and density in the non-African population (Table 3 and 4). This difference is also pronounced when comparing pathogenic and damaging SNPs (Figure 1 and 2).

## Discussion

At first glance the higher number and frequency of pathogenic SNPs in genomes from the non-African population can be explained by a relaxation of selection in the Out-of-Africa population [4,28,35]. However, three lines of evidence suggest that this is unlikely. Firstly, the opposite trend of SNPs in the damaging category (Table 3 and 4) suggests that, overall, the difference in strength of selection between the African and non-African populations is relatively minor. Second, data from the nuclear genome confirm our results that there is only a minor difference in selection between the African and non-African populations [4]. Finally, such subtle changes in selection pressure between African and non-African populations are expected to affect slightly deleterious alleles to a much larger extent than strongly deleterious alleles [4,36]. The pathogenic SNPs almost certainly belong to a more deleterious category of SNPs than all damaging SNPs and, therefore, relaxed selection cannot account for the observed differences in these two categories of SNPs between the African and the non-African populations.

The most parsimonious explanation for the observed pattern is a systematic ascertainment bias of pathogenic mutations leading to mitochondrial diseases in the non-African populations. Such a bias easily explains a higher number of pathogenic SNPs found in the non-African population (Figure 1) as well as their higher frequency relative to the damaging category (Figure 2). The presence of such a bias in genetic studies implies that we cannot get a full picture of the deleterious variability in the overall human population until such polymorphisms are comprehensively surveyed in African populations [35].

A wave of GWAS followed the suggestion that common diseases are caused by common pathogenic variants [16,17]. The present data show that knowledge of specific pathogenic variants from one population does not lead to a proportional discovery of pathogenic mutations in another, at least in the mitochondrial genome. Thus, it is likely that to advance the scope of personalized medicine the identification of pathogenic variants, especially in relation to GWAS, must be performed independently across all of human populations. Also, GWAS of specific human populations are likely to have more power for detecting disease-causing variants than studies with a sample of a mixture of humans from the total population.

## Conclusions

Our survey of the genome-wide variability in the mitochondrial human genome revealed three distinct patterns. First, selection against nonsynonymous alleles is stronger in the mitochondrial genome than in the nuclear one. Thus, the higher nucleotide diversity in the mitochondrial genome is likely explained by a higher mutation rate and not relaxation of selection. Second, a similar difference in the nucleotide density of all classes of SNPs implies that genetic drift is at present a stronger factor than selection in shaping differences in variability of the mitochondrial genome between African and non-African populations. Finally, the higher density of pathogenic SNPs in the non-African population is likely to be a result of an ascertainment bias in favour of discovering common pathogenic SNPs in the non-African population. Given the non-African focus of many GWAS [11,18,19] it is likely that this bias also affects our understanding of human pathologies with a nuclear-based genetic component.

## Competing interests

The authors declare that they have no competing interests.

## Authors' contributions

FAK and MSB contributed equally to the design, implementation and description of this study.

## Reviewers' comments

### Dr. Mikhail Gelfand, Department of Bioinformatics, Institute of Information Transfer Problems

*The authors present interesting, if straightforward, analysis, and the paper may be published more or less "as is", provided misprints and minor inaccuracies are corrected*.

*The only serious problem is the use of PolyPhen for the identification of damaging mutations. The PolyPhen analysis is based to a large degree on distant comparisons. But as the authors themselves have shown in one of their recent papers, a mutation that is damaging in a protein may well be observed in a distant protein. Hence PolyPhen should underestimate the underestimate the number of damaging mutations*.

This is a serious issue in the use of PolyPhen and this is the reason why we used only primate orthologs to call pathogenicity of SNPs in human genes. We believe, and it appears that the referee is in agreement with us, that this represents a much more careful approach that just the default use of PolyPhen.

*The other problem is that it is not obvious that it is correct to treat the African population as a homogeneous one. In fact, the non-African variation could be expected to be smaller simply because non-Africans are descendants of one branch of Africans*.

Yes, the overall variation in the non-African population is much lower that in the African one. The salt of our analysis is that when all variation is considered the African population is the most variable one, almost independent of the type of variation (synonymous, non-synonymous, etc). However, when variants that correspond to known pathogenic mutations are considered then the situation is reversed, the non-African population contains a larger number of such variants compared to the African population. The most parsimonious explanation for this pattern is that pathogenic mutations are to some extent population-specific and that there is a higher ascertainment of them in the non-African population.

### Dr. Vasily Ramensky, UCLA Center for Neurobehavioral Genetics, (nominated by Dr. Eugene Koonin)

*I have read the revised manuscript and would like to suggest publishing the current version provided that some minor typographic corrections are made. I appreciate the changes to the manuscript that I believe make the results of your work more straightforward and comprehensible*.

We thank the referee for taking the time to go through two rounds of the review process and for the helpful suggestions to improve our manuscript.

### Dr. David Rand, Department of Ecology and Evolutionary Biology, Brown University (nominated by Dr. Laurence Hurst)

This reviewer provided no comments for publication.
